# The Art of Self-Control – Autoregulation of Plant–Microbe Symbioses

**DOI:** 10.3389/fpls.2018.00988

**Published:** 2018-07-10

**Authors:** Chenglei Wang, James B. Reid, Eloise Foo

**Affiliations:** School of Natural Sciences, University of Tasmania, Hobart, TAS, Australia

**Keywords:** autoregulation, nodulation, arbuscular mycorrhizae, CLAVATA, CLE peptide, tomato

## Abstract

Plants interact with diverse microbes including those that result in nutrient-acquiring symbioses. In order to balance the energy cost with the benefit gained, plants employ a systemic negative feedback loop to control the formation of these symbioses. This is particularly well-understood in nodulation, the symbiosis between legumes and nitrogen-fixing rhizobia, and is known as autoregulation of nodulation (AON). However, much less is understood about the autoregulation of the ancient arbuscular mycorrhizal symbioses that form between Glomeromycota fungi and the majority of land plants. Elegant physiological studies in legumes have indicated there is at least some overlap in the genes and signals that regulate these two symbioses but there are major gaps in our understanding. In this paper we examine the hypothesis that the autoregulation of mycorrhizae (AOM) pathway shares some elements with AON but that there are also some important differences. By reviewing the current knowledge of the AON pathway, we have identified important directions for future AOM studies. We also provide the first genetic evidence that *CLV2* (an important element of the AON pathway) influences mycorrhizal development in a non-legume, tomato and review the interaction of the autoregulation pathway with plant hormones and nutrient status. Finally, we discuss whether autoregulation may play a role in the relationships plants form with other microbes.

## Introduction

Mycorrhizal symbioses between plants and fungi are widespread and ancient, with evidence from fossils and extant basal plants indicating that such interactions evolved during the colonization of land by plants between 450 and 475 mya (Field et al., 2015; Martin et al., 2017). The mycorrhizal fungi invade and extend the host plant’s root system, enabling enhanced nutrient uptake in exchange for fixed carbon. The most widespread are the arbuscular mycorrhizal (AM) associations that are characterized by the presence of a unique nutrient exchange unit, the fungal arbuscule. A significant amount of plant-derived carbon is invested, with estimates of between 4 and 20% of the carbon fixed by the plant transferred to the AM fungi (Bago et al., 2000; Douds et al., 2000). To prevent the energy cost to the plant outweighing the benefits of the interaction, plants might be expected to regulate AM colonization. Indeed, elegant physiological studies in flowering plants have revealed powerful systemic regulation of AM colonization. In split root studies, pre-colonization of one side of the root system can suppress subsequent colonization of the other side of the root system, providing evidence for a root–shoot feedback system termed autoregulation of mycorrhiza (AOM) (Vierheilig et al., 2000a, 2008; Meixner et al., 2005). Although there is no direct evidence that such control mechanisms also occur in more basal plant lineages, it is fair to assume that autoregulation would be an important element in the evolution of mutually beneficial plant–mycorrhizal interactions to prevent a potentially parasitic relationship developing.

Significant progress has been made in the past few decades in our understanding of the plant genes and signals that regulate AM symbioses, assisted greatly by the cross-over with the more recently evolved nodulation symbioses that occur between nitrogen-fixing rhizobial bacteria and (almost exclusively) legumes. In particular, the identification of a common symbiotic signaling pathway, required for the formation of both nodules and AM, has revealed that elements of the ancient AM pathway were co-opted into the more recently evolved nodulation pathway (Delaux et al., 2013; Martin et al., 2017). In particular, the common symbiotic pathway includes genes essential for initial communication between the plant host and rhizobia or AM fungi. However, a missing element in these comparisons has been the autoregulation of nodulation (AON) pathway. Like AM, nodulation is under powerful systemic control and the identity of AON signals and transduction pathways are now becoming clear (for review see Reid et al., 2011b; Soyano and Kawaguchi, 2014). Studies in legumes suggest some key cross-overs in the autoregulation pathways, as plant mutants disrupted in the AON pathway display not only hypernodulation but also hypermycorrhizal colonization (Morandi et al., 2000; Shrihari et al., 2000) and split root studies indicate nodulation can systemically suppress AM and vice versa (Catford et al., 2003). In this article, we examine our current understanding of AOM and begin to extend this beyond legumes by providing evidence of a role for the key AON gene in legumes, *CLAVATA2 (CLV2)*, in the AOM pathway of tomato. We also consider the role of plant hormones in autoregulation and examine the potential for the autoregulation of other beneficial plant–microbe interactions.

## Genes and Signals of the Autoregulation Pathway(S)

The systemic regulation of AM colonization in split root studies has been observed in both legumes and non-legumes (Vierheilig et al., 2000a; Meixner et al., 2007). This negative regulation appears to require a certain threshold of the amount of root colonized by AM (Vierheilig, 2004). AOM does not appear to be due simply to the strength of the carbon sink (Vierheilig et al., 2000b, 2008), but is rather regulated by a more specific pathway. Nodulation can systemically suppress AM and vice versa and even the application of Nod factors, the rhizobial-derived signaling molecules, can suppress AM (Catford et al., 2003), suggesting a clear overlap between the AON and AOM pathways. Similar conclusions were drawn from studies using a dual inoculation system in which both rhizobia and AM fungi were applied to the one root system (Sakamoto et al., 2013). This has also been explored in the non-legume barley, where it was found that inoculation with *Rhizobium* sp. could systemically inhibit AM formation. However, this inhibitory effect did not rely on Nod-factor production but was linked instead to the type 3 effector proteins of this rhizobial strain (Khaosaad et al., 2010).

**FIGURE 1:**
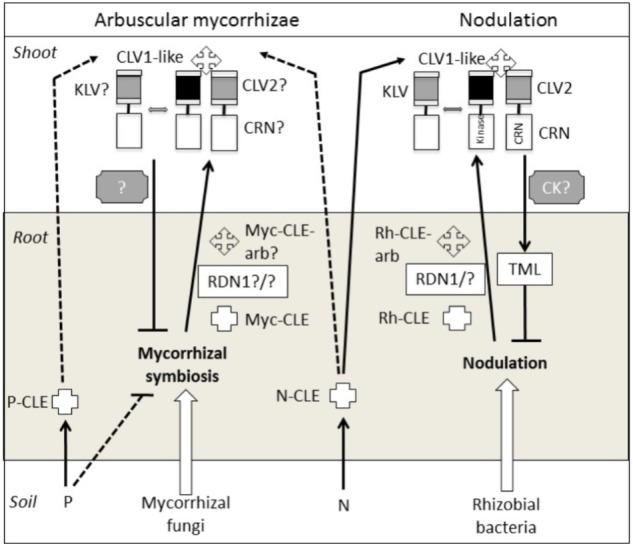
Proteins and signals that act in the shoot and/or root to autoregulate nodulation and mycorrhizal symbioses. Flat-ended lines indicate a negative influence, while arrows indicate a positive influence. Question marks and dotted lines indicate untested elements. CK, cytokinin; N, nitrogen; P, phosphorous; Rh, rhizobial; Myc, mycorrhizal; arb, arabinosylated.

A member of the Leucine-rich repeat (LRR) receptor kinase family that shares similarities with the CLAVATA1 (CLV1) protein (outlined below) has a central role in the AON in legumes (**Figure 1**). This *CLV1*-like gene is known as *NODULE AUTOREGULATION RECEPTOR KINASE (NARK)* in soybean, *HYPERNODULATION ABERRANT ROOT FORMATION 1 (HAR1)* in *Lotus japonicus*, *SUPER NUMERIC NODULES (SUNN)* in *Medicago truncatula* and *SYM29* in pea, and mutations in these genes result in hypernodulation (reviewed by Reid et al., 2011b). The CLV1-like protein appears to function as a shoot receptor for root-derived CLAVATA/ESR-related (CLE) peptides. In the root, events associated with nodulation generate specific rhizobial induced CLE peptides (Rh-CLE **Figure 1**) which in some cases appear to be arabinosylated via action of the enzyme ROOT DETERMINED NODULATION1 (RDN1) (e.g., *Mt*CLE12, *Gm*RIC1a, and *Gm*RIC1b) (Schnabel et al., 2011; Okamoto et al., 2013; Kassaw et al., 2017; Hastwell et al., 2018; Imin et al., 2018). The CLE peptides are then translocated to the shoot where they are perceived by a receptor complex that includes the CLV1-like protein (e.g., Okamoto et al., 2013). The perception of the root-derived signal(s) in the plant shoot generates a shoot to root signal that inhibits further nodulation. The shoot to root inhibitor is predicted to be a small molecular weight heat-stable molecule (Lin et al., 2010) that has been suggested to be cytokinin (Sasaki et al., 2014). Other elements of the AON pathway include the shoot acting CLV2 (Krusell et al., 2011), CORYNE (CRN) (Crook et al., 2016), and KLAVIER (KLV) (Miyazawa et al., 2010), all three of which encode LRR receptors that may also play a role in CLE perception, and TOO MUCH LOVE (TML), a root-acting F-Box protein that appears to act downstream of the shoot to root signal (Takahara et al., 2013).

In addition to its role in nodulation, the CLV1-like protein is also essential in the AOM pathway since *clv1-like* mutants across legume species (*sym29, sunn, nark*, and *har1*) also display hypermycorrhizal colonization (Morandi et al., 2000; Solaiman et al., 2000; Sakamoto and Nohara, 2009). It is important to note that the relatively small increase in AM in these mutant plants compared to wild type (WT) (between 20 and 50%) contrasts with the large (many fold) increase in nodule numbers of these lines compared with WT lines. Some have speculated that this relatively small increase may be due to the ability of the AM fungus to spread laterally in the root, meaning it is sometimes difficult to distinguish the effect of *clv1-like* mutations on subsequent AM infection (Schaarschmidt et al., 2013). One interpretation is that the AOM pathway may inhibit new AM infections at the epidermis but may not limit the spread of AM between cortical cells, although this has not been tested empirically. Such a hypothesis is consistent with the activation of AON during early stages of rhizobial infection (Li et al., 2009). Another way to examine the role of *CLV1-like* genes in AOM has been through the use of split root studies with mutant plants, which have shown that the *CLV1-like* gene in soybean (*NARK*) influences the suppression of subsequent mycorrhizal colonization (Meixner et al., 2005, 2007; Schaarschmidt et al., 2013). A role for NARK in the shoot control of AM was also observed in reciprocal grafts between the soybean *nark* mutant En6500 and WT plants (Sakamoto and Nohara, 2009) but not in a split root system with the same En6500 mutant (Meixner et al., 2007). Studies of orthologous mutants in other legumes species (e.g., *har1, sym29*) may be useful to clarify this inconsistency.

Apart from the requirement for the CLV1-like protein in AOM it is not yet clear if other AON genes encoding proteins that act in the root (RDN1, TML), shoot (CLV2, KLV, CRN) or as mobile signals (CLE) are also employed by the AOM pathway (**Figure 1**). One study examining the effect of a pea *clv2* mutant (*sym28)* found only a small but not significant increase in AM colonization compared with WT, although grafting and split root studies that would reveal if CLV2 plays a role in the systemic regulation of AM were not attempted (Morandi et al., 2000). This and other mutants disrupted in root and shoot acting elements of the AON pathway outlined above are available in a range of legumes, but to date their AM phenotypes and possible role in systemic AOM regulation has not been examined. Recent work in the legume *Lotus japonicas* indicated that mycorrhizal colonization and phosphate starvation generates CLE peptides distinct from those induced by nodulation or nitrogen (Funayama-Noguchi et al., 2011; Handa et al., 2015), but the function of these CLE peptides has yet to be tested. In an attempt to understand downstream elements of the AOM pathway, Schaarschmidt et al. (2013) analyzed the soybean transcriptome in split root mycorrhizal studies using WT and *nark* lines. Two putative CCAAT-binding transcription factor genes were identified, *GmNF-YA1a* and *GmNF-YA1b*, that are down-regulated by AM in a NARK-dependent manner. Hairy root RNAi lines with reduced expression of *GmNF-YA1a/b* displayed reduced AM colonization and this occurred in both WT and *nark* backgrounds, consistent with *GmNF-YA1a/b* acting downstream of *NARK* to suppress AM.

The evolutionary origin of the AON/AOM genes is still emerging but is informed by the *CLAVATA-WUSCHEL* (*CLV-*WUS) shoot meristem pathway. This pathway is best understood in Arabidopsis and involves several LRR receptors that act locally in the shoot, including CLV1, CLV2 and CRN, to perceive a CLE peptide, CLV3, which in turn activates a feedback loop to maintain a defined stem cell population in the shoot apical meristem (Hazak and Hardtke, 2016). Arabidopsis lines disrupted in these genes displayed altered shoot meristem formation. In pea and *Lotus japonicas*, mutant studies have revealed CLV2 plays a dual role, acting in both shoot development and AON, as *clv2* mutants display hypernodulation and shoot fasciation (Krusell et al., 2011). In contrast, there appear to be specific CLE genes that act in the AON pathway (Mortier et al., 2010). Similarly, disruption of *CLV1-like* genes closely related to *CLV1* in pea, *Lotus*, *Medicago* and soybean (*SYM29, HAR1, SUNN*, and *NARK*, respectively) result in hypernodulation but not shoot fasciation (reviewed by Reid et al., 2011b). A possible explanation in soybean for divergence between *NARK* and *CLV1*, is that *CLV1* appears to have undergone a duplication, resulting in *NARK* and *CLV1A* (Yamamoto et al., 2000) but this does not appear to be the case in the other legumes examined (see Schnabel et al., 2005). *CLV1A* is more closely related to *AtCLV1* and recent studies have shown it influences shoot architecture but not nodulation (Mirzaei et al., 2017). Although many phylogenetic studies have examined the *CLV1, CLV2* and *CLE* gene families, these studies have been almost exclusively limited to angiosperms (e.g., Sun and Wang, 2011; Zan et al., 2013; Wei et al., 2015; Xu et al., 2015; Hastwell et al., 2017), preventing a more comprehensive understanding of the evolutionary history and possible functional diversification of these genes. One recent transcriptomic study failed to find evidence for the *CLAVATA-WUSCHEL* (*CLV-*WUS) pathway in the moss *Physcomitrella* and liverwort *Marchantia* (Frank and Scanlon, 2015). However, a more comprehensive examination of these gene families in basal plant lineages and in mycorrhizal vs. non-mycorrhizal species (see approach of Favre et al., 2014; Delaux et al., 2015) might provide an insight into their possible role in the AM program.

## Role of Clv2 in Aom of the Non-Legume Tomato

To fully understand the genes, signals and evolutionary history of AOM, we must go beyond legumes. Indeed, a non-legume system removes the possible complication of cross-talk between the AON and AOM pathways to allow us to identify AOM components. Therefore, we employed the tomato *clv2-2* line, a CRISPR-Cas9 knock out line that targets the *CLV2* gene (Xu et al., 2015). As outlined above, this gene is essential for AON in legumes and also acts to control shoot apical meristem formation in legumes (Krusell et al., 2011) and non-legumes, including *clv2* tomato lines that display a weakly fasciated shoot and an increase in the number of floral organs (Xu et al., 2015).

**FIGURE 2:**
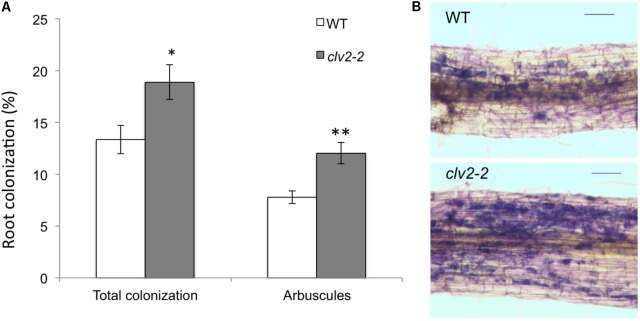
Mycorrhizal colonization in tomato wild type (WT, M82) and *clv2-2* lines. **(A)** Percentage of the root colonized by all fungal structures and arbuscules. *n* = 12, values are means + SE *t*-tests were performed for each parameter, ^∗^*P* < 0.05, ^∗∗^*P* < 0.01. **(B)** Photograph of a typical length of colonized root (scale bar = 100 μm) after staining fungal structures (blue). Tomato seeds were germinated in potting mix for 2 weeks and 12 equal sized seedlings of each genotype were selected and transplanted to 2 L pots. The pots were premixed with vermiculite and gravel (1:1) plus mycorrhizal inoculum (1/5 volume of corn roots colonized with *Rhizophagus irregularis* and associated potting medium) and topped with vermiculite. The seedlings were grown in a glasshouse with the following condition: 18 h photoperiod, 25°C day/20°C night. Plants were nutriented three times a week with modified Long Ashton solution (5 mM N and 0.5 mM P) (Hewitt, 1966). Plants were harvested 6 weeks after transplanting. The root was cut into 1 to 1.5 cm segments and stained using the ink and vinegar method (Vierheilig et al., 1998). 25 root segments were selected per plant and mycorrhizal colonization scored using the intersect scoring method (McGonigle et al., 1990). Blind labeling was used to avoid any potential bias during the scoring process.

We tested if the *CLV2* gene plays a role in AM development by examining the AM phenotype of the tomato *clv2-2* line. Compared with WT, *clv2* plants displayed a significant increase in AM colonization, including arbuscule frequency, compared with WT plants (**Figure 2A**). Although more frequent, the mycorrhizal structures observed in *clv2* mutants including arbuscules were similar in appearance to WT (**Figure 2B**). Therefore, we provide the first evidence that CLV2 in tomato, known to be important for AON in legumes, also acts as a negative regulator of AM. This provides the first genetic evidence for the AOM pathway in non-legumes.

## Role of Plant Hormones in Aom

Autoregulation of mycorrhizae and AON are regulated by systemic signals and in addition to mobile CLE peptides, a range of studies have examined the role of plant hormones in AON and in some cases AOM. Double mutant studies in pea indicate gibberellins, brassinosteroids, and strigolactones are not required for the supernodulation phenotype and thus do not appear to act downstream of AON elements, CLV1-like, CLV2 or RDN1 (Ferguson et al., 2011; Foo et al., 2014b). In contrast, transcriptional studies showed that either jasmonic acid (JA) biosynthesis genes or JA regulated genes were systemically regulated by rhizobial colonization, and this was mediated by GmNARK in soybean. These results suggest the AON pathway influences JA signaling (Kinkema and Gresshoff, 2008). Several studies have also examined a role for auxin in autoregulation. In split root studies in soybean, significant auxin accumulation was observed in AM colonized roots but not uncolonized roots. However, this increase was not as great in the roots of a *nark* mutant (Meixner et al., 2005). This contrasts with nodulation studies with the orthologous mutant in *Medicago, sunn*, that suggest SUNN may be required to suppress auxin accumulation in the root following rhizobial challenge (van Noorden et al., 2006). In WT *Medicago*, challenge with rhizobia leads to the downregulation of auxin transport from the shoot to the root. In contrast, *sunn* mutants displayed elevated auxin levels in the infection zone of the root following inoculation with rhizobia. These studies suggest the AON pathway may modulate shoot to root auxin transport but this has yet to be investigated directly in AOM.

Cytokinin is an interesting case as it has been suggested to be a candidate for the shoot-derived inhibitor in AON, based on several lines of evidence from plants with altered cytokinin or CLE peptide biosynthesis, and measurement of cytokinin levels and transport (Sasaki et al., 2014). However, a key finding of this paper, that the LORE *ipt3-2* mutant allele, which is disrupted in a key cytokinin biosynthesis gene, has increased nodulation could not be repeated in an independent study (Reid et al., 2017). In addition, another study indicated cytokinin may promote nodulation via the AON pathway. In *Medicago*, application of cytokinin directly to roots could induce the expression of the *MtCLE13* gene (Mortier et al., 2012) believed to encode the root to shoot AON signal. Given the ability for nodulation to suppress AM and vice versa outlined above, it is likely that the shoot-derived inhibitor is a common signal between the AON and AOM pathways. Studies with grafts between legume species certainly suggest that the shoot-derived inhibitor of nodulation is conserved across species (e.g., Lohar and VandenBosch, 2005; Ferguson et al., 2014; Foo et al., 2014a). However, unlike its clear role in nodulation there is less evidence to suggest cytokinin has an influence on AM. For example, the cytokinin receptor mutant in *M. truncatula*, *cre1*, did not display any alteration in AM development (Laffont et al., 2015). However, pharmacological studies suggest that cytokinin may promote AM development in pea (F. Guinel, personal communication). Clearly, questions remain around the role of CK in AON, in particular as the shoot-derived inhibitor, and studies directly testing its endogenous role in AOM are required.

## Do Nutrient Status and Other Beneficial Plant–Microbe Symbioses Interact With the Autoregulation Pathway(S)?

Forming symbioses with rhizobial or AM partners may only be beneficial to the plant under conditions of low mineral nutrient availability. In particular, legumes severely reduce nodulation when roots are exposed to elevated nitrogen levels and there are important roles for elements of the AON pathway in this nitrate-response (see Reid et al., 2011b). For example, the *clv1-like* mutants across legumes display a reduced ability to suppress nodulation in response to nitrate (Schnabel et al., 2005; Lim et al., 2011; Okamoto and Kawaguchi, 2015). This reduced response to nitrogen is also seen in *klv* and *rdn1* mutants (e.g., Jacobsen and Feenstra, 1984; Oka-Kira et al., 2005). However, this has not been comprehensively examined for all AON mutants across species. In addition, nitrate treatment induces the expression of specific CLE peptides, which in some cases are the same as those that are induced by rhizobia (Okamoto et al., 2009; Reid et al., 2011a). Whether the AOM pathway plays any role in nitrogen-regulation of AM symbioses has not been explored and, unlike the clearly suppressive effects on nodulation, it is not even clear if nitrogen is a promoter or inhibitor of AM (Correa et al., 2015). In contrast, phosphate has a strong suppressive influence on AM and this influence is systemic and does not require strigolactones (Breuillin et al., 2010; Foo et al., 2013b). Although phosphate induces expression of specific CLE peptides (Funayama-Noguchi et al., 2011), there is no direct evidence that the AOM pathway mediates the phosphate response of AM. Indeed phosphate regulation of AM is maintained in soybean and pea mutants disrupted in the *clv1-like* mutants, *nark* and *sym29* (Wyss et al., 1990; Foo et al., 2013a). However, phosphate positively regulates nodule number and studies in pea have found this is disrupted in the *sym29* mutant (Foo et al., 2013a), suggesting a cross-over in the AON and phosphate response pathways. MicroRNAs of the 399 family have also been shown to play a role in the phosphate response and some were shown to be induced by phosphate-starvation in AM colonized *Medicago* (Branscheid et al., 2010). However, as overexpression of these miR399 genes in tobacco did not influence AM colonization, no clear role for these mobile microRNAs were established in the AM phosphate response (Branscheid et al., 2010).

In addition to nodulation and AM, plants form a range of other beneficial interactions with soil microbes. These include actinorhizal symbioses between members of the fabid clade and *Frankia* bacteria, ectomycorrhizal symbioses and interactions with fungal and bacterial endophytes. Systemic regulation of colonization has been demonstrated for actinorhizal associations (Wall and Huss-Danell, 1997), and for the interaction between Arabidopsis and the fungal enodphyte *Piriformospora indica* (Pedrotti et al., 2013). Indeed, it has been shown in some split root studies that plants infected with endophytes have a decreased level of AM colonization, although this was not found in all studies (Müller, 2003; Omacini et al., 2006; Mack and Rudgers, 2008). Phylogenetic studies have suggested that the common symbiotic pathway is conserved in AM, rhizobial and actinorhizal associations, although the role of these genes in ectomycorrhizae and endophyte relationships is not known (Martin et al., 2017). Arabidopsis is a particularly interesting case as it appears to have lost the majority of the common symbiotic pathway (Delaux et al., 2014), consistent with the lack of AM colonization and suggesting that this pathway is not important for hosting fungal endophtyes. However, as these studies did not include AON/AOM genes the evolutionary origin of these pathways and their potential role across species is still not clear.

## Conclusion and Future Perspectives

The similarities between the AOM and AON pathways and their shared genetic components is consistent with the AON pathway evolving at least in part from a pre-existing AOM pathway in early land plants. However, a lack of phylogenetic, genetic and physiological studies in non-legumes, including basal land plants, has hampered our understanding of the origin and diversification of the autoregulation pathway. In this paper, we show that in the non-legume tomato, the *CLV2* gene suppresses AM development, providing the first genetic evidence for an AOM gene in a non-legume. As found in legumes, this gene also plays a role in shoot apical meristem maintenance. However, the precise delineation in function of other AON/AOM elements such as the *CLV1* and *CLE* genes in shoot apical meristem maintenance is still not clear. Furthermore, it is likely that multiple systemic pathways regulate symbioses (Kassaw et al., 2015). For example, novel peptides and accompanying perception pathways with roles in nodulation and root development are now emerging (e.g., CEP1 and CRA2, Mohd-Radzman et al., 2016). Future studies could more systematically examine the role of AON genes and peptide signals in AM development and take a phylogenetic approach to examine the evolutionary origin of symbiotic autoregulation.

## Author Contributions

EF conceived the project. CW carried out experiments. CW, JR, and EF wrote the manuscript.

## Conflict of Interest Statement

The authors declare that the research was conducted in the absence of any commercial or financial relationships that could be construed as a potential conflict of interest.
